# Preeclampsia Screening

**DOI:** 10.3390/diagnostics16132074

**Published:** 2026-07-02

**Authors:** Yunyu Chen, Liona C. Poon

**Affiliations:** Department of Obstetrics and Gynaecology, Prince of Wales Hospital, The Chinese University of Hong Kong, Hong Kong SAR, China

**Keywords:** first trimester, preeclampsia, prediction, screening, second trimester, third trimester

## Abstract

Preeclampsia is a leading cause of maternal and perinatal morbidity and mortality worldwide. This significant burden necessitates effective early identification of pregnancies at high-risk for preeclampsia. Accurate prediction is essential in order to develop and optimize preventive strategies. The evolution of preeclampsia screening has progressed from a traditional checklist-based approach to individualized, multivariable models. The first-trimester triple test, which was developed by the Fetal Medicine Foundation (FMF), represents this advancement. It utilizes Bayes’ theorem to calculate patient-specific risks by integrating maternal factors, mean arterial pressure, uterine artery pulsatility index, and serum placental growth factor. This model, called “first trimester FMF triple test”, has undergone successful internal and external validation for the prediction of preterm preeclampsia. To ensure the reliability of biomarker measurements and achieve an optimal screening performance, it is essential to implement standardized measurement protocols and rigorous quality control processes in biomarker testing. The triple test could also be utilized in the 2nd and 3rd trimester, and the addition of biomarkers such as soluble fms-like tyrosine kinase-1 further improves risk stratification assessment and continued surveillance of high-risk pregnancies.

## 1. Introduction

As a multi-system pregnancy disorder, preeclampsia is characterized by the new- onset hypertension with significant proteinuria after 20 weeks of gestation [1]. Affecting 2–8% of pregnant women, preeclampsia is one of the leading causes of maternal and perinatal morbidity and mortality, responsible for 76,000 maternal and 500,000 fetal deaths worldwide [2]. Its immediate maternal adverse effects include injuries to the hepatorenal and coagulation systems. In severe or untreated cases, it may lead to life-threatening conditions such as maternal pulmonary edema, eclampsia, brain injury, and death [2]. For the fetus, inadequate uteroplacental perfusion may result in fetal growth restriction (FGR) and or placental abruption, thereby leading to indicated premature birth or stillbirth. Additionally, the consequences of PE are long-lasting, including an increased risk of long-term cardiovascular diseases in both the mothers and their offspring from the affected pregnancy [3]. Specifically, women with preeclampsia are at greater risk of chronic hypertension, stroke, metabolic syndromes, cognitive impairment, and chronic end-stage renal disease later in life [4,5]. The offspring of preeclamptic pregnancies carry a higher risk of subsequent complications such as neurodevelopmental impairment, insulin resistance, diabetes mellitus, coronary heart disease, and hypertension [5].

Screening women for high-risk of preeclampsia in the first trimester allows for low-dose aspirin prophylaxis, thereby enhancing outcomes for mothers and their babies [6]. Screening for preeclampsia in the second and third trimesters enables close monitoring, timely intervention, and planned delivery for high-risk individuals. In recent years, multiple algorithms have been developed and refined to screen for preeclampsia risk during pregnancy. This review aims to outline methods for screening preeclampsia for optimization of patient care across all stages of pregnancy.

## 2. First Trimester Screening

The subclinical first stage of preeclampsia typically occurs in the first trimester of pregnancy, which is considered a window of opportunity for early screening to identify women at high risk of preeclampsia. For these high-risk individuals, early preventative measures may help enhance placentation, thereby reducing related morbidities and mortalities. In addition, more intensive antenatal monitoring for high-risk women allows for earlier detection of preeclampsia and timely management. It has been proposed to categorize preeclampsia according to gestational age at delivery as follows: early-onset preeclampsia (with delivery at <34 + 0 weeks’ gestation), preterm preeclampsia (with delivery at <37 + 0 weeks’ gestation), late-onset preeclampsia (with delivery at ≥34 + 0 weeks’ gestation), and term preeclampsia (with delivery at ≥37 + 0 weeks’ gestation) [7]. Early-onset or preterm preeclampsia is associated with an increased risk of maternal and perinatal morbidity and mortality compared to late-onset or term preeclampsia [8]. As there is no single test that could effectively determine the risk of preeclampsia, various algorithms have been developed and refined in recent years to enhance the screening performance for preterm preeclampsia. However, the first-trimester screening approach has limited utility for the prediction and prevention of term or late-onset preeclampsia [9].

### 2.1. Maternal Risk Factors

#### 2.1.1. A List of Maternal Risk Factors for Preeclampsia

A checklist of factors in maternal history has been published following guidelines in The National Institute for Health and Care Excellence (NICE) of the United Kingdom for assessment of the risk of preeclampsia [10]. However, this simple checklist has been shown to achieve detection rates of 89.2%, 93.0%, and 85.0% for the prediction of early and late preeclampsia and gestational hypertension, respectively, at a 64.1% of false positive rate (FPR) [11]. The NICE subsequently refined its 2010 guidelines, stratifying maternal factors into “moderate” and “high risk” for preeclampsia [12]. Following the NICE guidelines, the presence of one high-risk factor (hypertensive disease in previous pregnancy, chronic renal disease, chronic hypertension, diabetes mellitus (or autoimmune disease), or two or more moderate-risk factors (nulliparity, age ≥ 40 years, body mass index (BMI) ≥ 35 kg/m^2^, interpregnancy interval of >10 years or family history) are considered high-risk for preeclampsia. For these individuals screened at high risk of preeclampsia, 75 mg of aspirin daily is recommended, starting from 12 weeks of gestation until delivery. These recommendations were updated in 2019 to specify that the dose of aspirin should be 75–150 mg daily from 12 weeks of gestation until the birth of the baby, and to include multiple pregnancy as a moderate-risk factor.

#### 2.1.2. Relevant Guidelines and Screening Performance

Following the American College of Obstetricians and Gynecologists (ACOG)report on risk factors of preeclampsia [13], the United States Preventive Services Task Force (USPSTF) recommended low-dose aspirin prophylaxis for the prevention of preeclampsia for women with one or more high-risk factors or more than one moderate-risk factor in 2014. In 2018, ACOG endorsed the United States Preventive Services Task Force guidelines: low-dose aspirin (81 mg/day) should be initiated for women with one or more high-risk factors or two or more moderate-risk factors, starting from 12 to 28 weeks (preferably before 16 weeks) until delivery [13]. The risk-based strategy for aspirin prophylaxis is endorsed by other international bodies, including the International Society for the Study of Hypertension in Pregnancy (2018) [14], the International Federation of Gynecology and Obstetrics (FIGO) (2019) [7], and the International Society of Ultrasound in Obstetrics and Gynecology (ISUOG) [14].

#### 2.1.3. Clinical Impact of Relying on a Maternal Risk Factor for Preeclampsia Screening

A checklist-based approach is largely based on retrospective epidemiological studies on the associations between the risk factors and the development of preeclampsia. The development of new prediction models has challenged the effectiveness of NICE and ACOG screening guidelines. A large study, including 8775 singleton pregnancies at 11–13 weeks of gestation, demonstrated that detection rates of NICE-based screening recommendations were only 39% and 34% for predicting preterm and term preeclampsia, respectively, at a FPR of 10.2% [11]. Nevertheless, the majority of studies did not distinguish risk factors based on the severity of the disorder or differentiate preterm from term cases [15].

Overall, the simple checklist screening of maternal risk factors is practical for identifying women at risk, but remains insufficient for effective prediction of preeclampsia [11]. Therefore, there is an essential need to improve the prediction performance of preeclampsia by utilizing a personalized approach. In recent years, a growing body of evidence highlights the potential of combining biophysical and biochemical biomarkers into an individualized screening strategy, moving beyond traditional checklist-based methods for preeclampsia screening.

### 2.2. Biophysical Markers

#### 2.2.1. Mean Arterial Pressure

Women who subsequently develop preeclampsia have been shown to exhibit elevated blood pressure (BP) as early as the first and second trimesters of pregnancy [16].

**Figure 1:**
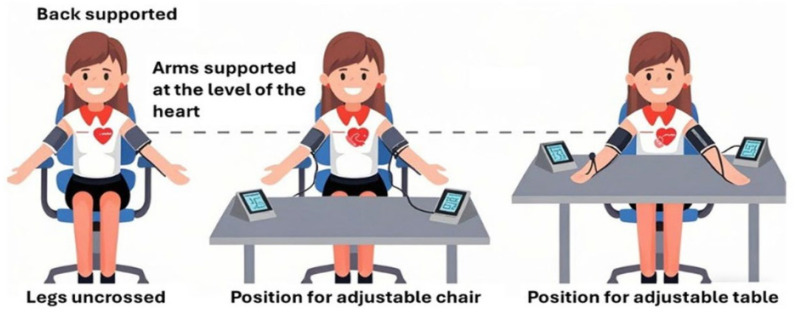
The standardized position for blood pressure measurement. This figure is modified with acknowledgements from [8].

Standardized BP measurement is important for effective preeclampsia screening. Inconsistencies in measurement techniques or deviations from the protocol may compromise the accuracy of screening performance. According to the FIGO guideline for first-trimester screening of preeclampsia [7], women should rest for five minutes prior to BP measurements. Women are seated with their backs supported, their arms resting at the level of the heart, and their legs flat on the floor. The correct cuff size is determined by measuring the mid-arm circumference: small (<22 cm), normal (22–32 cm), or large (33–42 cm) (Figure 1). BP is measured for both arms simultaneously. This will yield two sets of BP recordings; more precisely, four sets of systolic BP and diastolic BP measurements at 1 min intervals. The MAP measurement is derived by averaging these four sets of measurements. Additionally, there is a debate on which arm (left arm or right arm) to use for BP measurement. Data from Poon et al. demonstrated that 8.3% of normal pregnant women (*n* = 5435) exhibited a significant BP difference between the two arms (defined as an inter-arm difference of >10 mmHg of systolic BP and diastolic BP) [17]. These observations suggest the importance of measuring BP in both arms.

Multiple factors, such as gestational age, BMI, maternal age, ethnicity, smoking, family history of preeclampsia, history of previous preeclampsia, diabetes mellitus, and chronic hypertension, affect MAP measurements [18]. Consequently, MAP should be standardized by converting to a multiple of the median (MoM). The MoM represents the degree of deviation of the result of the test from the median of the normal population (the expected value in the normal population). The expected value is calculated using a formula from a multivariable regression analysis, which accounts for all independent variables of the biomarker.

#### 2.2.2. Uterine Artery Pulsatility Index

Abnormal uterine artery Doppler velocimetry in the first trimester was shown as an early sign of impaired uteroplacental circulation. Mean UtA-PI decreased in the first trimester, and it has been shown to be associated with preterm preeclampsia but not with term preeclampsia, with AUC of 0.69 (95% CI: 0.57–0.80) and 0.52 (95% CI: 0.48–0.56), respectively [19]. A meta-analysis involving 27 studies reported that uterine artery pulsatility index (UtA-PI) achieved a sensitivity of 0.59 at a 12% false positive rate and a specificity of 0.879 for the prediction of preeclampsia, and UtA-PI measurements obtained before 20 weeks did affect the screening performance of preeclampsia [20].

**Figure 2:**
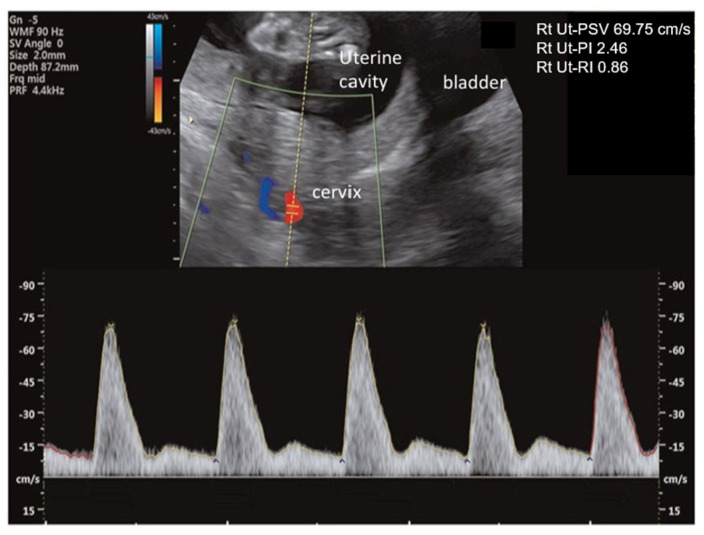
Measurement of uterine artery resistance indices via sagittal view of the cervix in the first trimester.

A standardized measurement of UtA-PI is important to ensure the reproducibility, consistency, and accuracy of preeclampsia screening. To ensure consistency, UtA-PI measurements at the level of the cervical internal os are recommended to be obtained via transabdominal ultrasound (Figure 2). The first trimester UtA-PI measurements at the internal os level have been shown to be more reproducible than measurements acquired at the crossover of the external iliac vessels level [21]. Additionally, a stronger correlation in the UtA-PI between the first and second trimester measurements was observed when the first trimester measurement was acquired at the internal os level rather than at the crossover of the external iliac vessels [21]. UtA-PI measurements on a midsagittal view demonstrate moderate intra- and inter-observer reproducibility. The inter-observer reliability shows moderate to good reproducibility when performed by experienced operators (ICC: 0.92), whereas the agreement among less experienced operators is not satisfactory (ICC: 0.74). These findings suggest that inter-observer reproducibility is influenced by the operator’s experience, highlighting the importance of adequate training for operators in UtA PI measurement [22,23,24]. There is evidence suggesting that the transverse plane for UtA-PI measurements provides greater reliability, reproducibility, and takes less time, while being potentially easier compared to the sagittal plane [25]. Given that existing prediction models were developed based on the sagittal plane, the transverse plane could be a complementary approach for the measurement of UtA-PI.

Several factors, including maternal age at the birth of the last pregnancy, gestational age, and birthweight Z-score, have been reported to affect the measurement of UtA-PI [15]. Consequently, to compare the preeclampsia group and the unaffected group, adjustment for these variables and conversion into MoM shall be performed.

#### 2.2.3. Maternal Biochemical Markers

The most informative biochemical biomarkers for predicting preeclampsia are the angiogenic and anti-angiogenic factors, including placental growth factor (PlGF), soluble fms-like tyrosine kinase-1 (sFlt-1), and soluble endoglin (sEng) [26,27,28]. Maternal serum sFlt-1 levels rose around five weeks preceding the development of preeclampsia. However, from 13 to 16 weeks of gestation, maternal PlGF levels were significantly reduced in women who developed preeclampsia compared to controls, with the greatest difference observed weeks prior to the clinical diagnosis of preeclampsia. Later research by Romero et al. further detailed these profiles among women with preterm or term preeclampsia and small for gestational age (SGA) [29]. The key findings included: (1) lower maternal plasma PlGF concentrations from 10 to 11 weeks of gestation onwards, in both preeclampsia (preterm or term) and SGA pregnancies; (2) increased maternal plasma sFlt-1 concentrations in women who developed preterm and term preeclampsia (*p* = 0.009, and 0.020, respectively) at 26 and 29 weeks of gestation, but no significant difference in those with SGA when compared to controls (*p* = 0.147 at 25 weeks and *p* = 0.8285 at 40 weeks) [30]; (3) higher maternal plasma sEng level from 10, 23, and 30 weeks in pregnancies affected by preterm, term preeclampsia and SGA, compared to controls. These findings suggest that PlGF may hold promise in identifying the risk of preeclampsia from the first trimester. In contrast, sFlt-1 and sEng appear more relevant later in pregnancy (in the second or third trimester onwards) [29]. Motivated by these findings, angiogenic and anti-angiogenic factors have been incorporated for risk assessment and prediction of preeclampsia in the first trimester and before clinical presentations become apparent. Additionally, significant inter-manufacturer differences exist in the performance of available PlGF immunoassays, particularly regarding assay sensitivities/recovery and cross-reactivity. Therefore, measurements obtained from different platforms are not interchangeable, and each assay necessitates independent validation against clinical outcomes [30]. There is limited evidence supporting the use of PlGF measured before 10 weeks’ gestation. A prospective study including 8386 pregnant women with blood samples taken at 8–14 weeks’ gestation, demonstrated that there were no significant differences in PlGF MoM between women with PE and those with unaffected pregnancies in blood samples obtained before 10 weeks’ gestation. It provides evidence that the timing for PlGF sampling might be adjusted from 11 to 14 weeks to 10–14 weeks’ gestation, as lower PlGF MoM in pregnancies affected by preterm preeclampsia was observed than in unaffected pregnancies when samples were obtained at 10 weeks’ gestation [31]. Regarding the comparison of the performance of PlGF sampling at 10–14 weeks and 11–14 weeks for the prediction of preeclampsia, Rode et al. reported that the addition of PlGF measured at or after 10 weeks to maternal factors increased the detection rate from 31.3% to 56.3% at a 10% screen positive rate for predicting preterm preeclampsia. Adding PlGF measured at 11–14 weeks to maternal factors, MAP, and UtA-PI, enhanced the performance of preterm preeclampsia screening, increasing the detection rate from 50.9% to 67.3% at a 10% of screen positive rate [32].

In addition, decreased circulating pregnancy-associated plasma protein A (PAPP-A) at 11–14 weeks has been shown to be associated with the development of PE by reducing the availability of unbound insulin-like growth factor, thereby limiting their cellular function necessary for placental growth and development. A systematic review and meta-analysis demonstrated that maternal PAPP-A levels below the 5th percentile are associated with an increased risk of PE (OR: 1.94; 95% CI, 1.63–2.30). However, the predictive value is limited, with a detection rate of 16% (95% CI 9–28%) at 8% FPR for the prediction of preeclampsia [33]. Furthermore, the timing of blood sampling does not significantly affect the predictive value of PAPP-A for preeclampsia in the first trimester [32]. A recent study including 8386 women who underwent routine first-trimester aneuploidy screening at 8–14 weeks of gestation, reported that the addition of PAPP-A measured before 10 weeks to maternal factors alone, or maternal factors, MAP, and UtA-PI, does not significantly improve the performance for the prediction of preterm preeclampsia [32].

Similar to MAP and UtA-PI, the PlGF and PAPP-A levels are affected by maternal and pregnancy characteristics. To ensure consistency, PlGF and PAPP-A measurements should be expressed as MoM values, adjusting for gestational age, maternal factors such as weight and racial origin, associated with individual biomarkers, analyzer as well as reagents used [34].

#### 2.2.4. Preeclampsia Prediction Algorithms

##### Model Development

The initial FMF prediction model for preeclampsia was developed from a prospective study of 7797 singleton pregnancies, including 157 cases with preeclampsia [35]. Using multivariate analysis, incorporating maternal factors, MAP, UtA-PI, serum pregnancy-associated plasma protein A (PAPP-A), and PlGF, measured at 11–13 weeks of gestation, namely “the first trimester triple test”, this test achieved detection rates of 93% for the prediction of early-onset preeclampsia and 36% for the prediction of late-onset preeclampsia, respectively, at a 5% FPR, which was superior to the performance using the traditional checklist-based method, including maternal factors only [35].

The competing risk model was originally developed using data from 58,884 singleton pregnancies at 11–13 weeks of gestation, among which 1426 (2.4%) subsequently developed preeclampsia. At a fixed 10% FPR, the model yielded detection rates of 77% and 54% for predicting preterm preeclampsia and any-onset of preeclampsia, respectively [36]. A subsequent update of the original algorithm, using data from 35,948 singleton pregnancies, with 1058 pregnancies (2.9%) with preeclampsia, reported the detection rates of 75% and 47% for the prediction of preterm and term preeclampsia, respectively, at a FPR of 10% [37]. The largest study, by Tan et al. (2018), included data from three reported non-intervention screening studies at 11–13 weeks’ gestation among 61,174 mixed-European singleton pregnancies, among which 1770 women subsequently developed preeclampsia (2.9%) [34]. This study demonstrated that a combination of maternal factors with MAP, UtA-PI, and serum PlGF (namely “the FMF triple test”) provided the best predictive performance in detecting 75% of cases with preterm preeclampsia and 41% of cases with term preeclampsia, respectively, at a FPR of 10% [34].

There are several cohorts that developed a logistic regression-based model [38,39,40]. However, there are some limitations of the logistic regression approach, including its treatment of PE as a binary outcome and the need for separate models for different types of PE. Additionally, the logistic regression model lacks flexibility, as the inclusion of new biomarkers would require the development of a new model [18]. A systematic review and meta-analysis reported poor-to-good discrimination performance (median AUC, 0.66 (range, 0.53–0.77)) and overfitted calibration when using logistic regression models for predicting preterm PE on external validation [41]. In addition, none of the models has achieved successful external validation.

Subsequent models have also been developed using data from the Spanish population. Scazzocchio et al. (2013) developed a combined model incorporating maternal factors, mean UtA-PI, and MAP [42]. While this group reported its performance of the internally validated study in 2017 [42], other subsequent studies revealed suboptimal performance upon external validated studies [43,44]. A separate 2015 Spanish algorithm incorporated additional biomarkers, including PlGF and sFlt-1 [45], but an external validation in a Dutch population showed suboptimal calibration [43]. Another Spanish research group proposed a model based on a multivariable Gaussian distribution, suggesting potential adaptability across populations [46]. Despite these efforts, a key limitation shared by these models is that they were developed on a small number of cases, lacking sufficient internal and external validation.

##### Validation of Prediction Models for Preeclampsia

An effective prediction model should be evidence-based and relevant to the population. It is well established that the effectiveness of screening is affected by the incidence of preeclampsia, maternal characteristics, and biomarker variability. Furthermore, since the model performance generally decreases when applied outside the original population, external validation is essential before clinical implementation of prediction models has been established. External validation, considered the best method for model evaluation, assesses a model’s generalizability by testing its performance on independent cohorts from distinct but ‘plausibly related’ populations.

##### Validation of the First Trimester Fetal Medicine Foundation Preeclampsia Prediction Models

Although effective first-trimester prediction models have been developed to inform appropriate preventive strategies, their applicability is limited as the model development may be derived from specific populations and clinical settings. Hence, their predictive performance may be limited when applied to other populations, as it could be influenced by variations in population characteristics, such as racial origin, height, weight, and the prevalence of preeclampsia. External validation is necessary to assess a prediction model’s reproducibility and generalizability to different settings or populations [47,48].

The performance of validation studies for the preeclampsia prediction model has yielded divergent results. For instance, the results of a UK validation of the FMF triple test were comparable to those from the original study [49]. The first trimester prediction model for preeclampsia by Poon et al. [35] stands out for its successful validation across diverse populations, including those in the United States [50], Australia [51], Italy [52], Brazil [53,54], and multiple combined European populations [55]. In contrast, there is also evidence reporting discrepancies in predictive performance of the first-trimester prediction model of preeclampsia between validated and original studies [56]. Despite these inconsistencies, a multi-center study in 10,935 pregnancies across seven regions in Asia by Chaemsaithong et al. (2020) confirmed the effectiveness of the Bayes’ theorem-based FMF triple test at 11–13^+6^ weeks gestation, achieving a 64.0% detection rate for preterm preeclampsia at a fixed FPR of 10% [18]. The FMF triple test is the only model that has undergone extensive and successful internal and external validations. Subsequently, this test was endorsed by the International Society of Ultrasound in Obstetrics and Gynecology (ISUOG) in 2018 and the International Federation of Gynecology and Obstetrics (FIGO) in 2019 [7,14].

#### 2.2.5. Quality Assessment

To ensure optimal screening performance, it is important to establish standardized protocols for biomarker measurements and quality control, as each biomarker is inherently susceptible to measurement inaccuracies, thereby compromising screening performance. Quality assessment is essential for preeclampsia screening, as biomarkers are prone to measurement variations when protocols are poorly adhered to. Changes in the batch of reagents used, temperature shifts [57], and deviation from the manufacturer’s instructions may lead to inaccurate biochemical marker results. A process for quality control must be performed regularly to ensure data standardization, reliability, and accuracy. Any deviation in screening values should be promptly investigated to identify the causes, and necessary corrections should be made promptly.

##### Screening Process—The Fetal Medicine Foundation Triple Test

External validation of the predictive performance of the FMF triple test in the local population and continuous quality control is important to ensure its successful clinical implementation. Its clinical implementation involves components, which vary in complexity and general applicability, and the costs of the different components. The steps for the FMF triple test are outlined as follows.

(1) Maternal factors, including demographics, medical and obstetric history, weight, and height, are recorded into the risk calculator using software or documented by trained staff with a standardized case report form. For the latter, the data are then transcribed into the risk calculator. Data quality is maintained through periodic audits employing quality assurance tools. Additionally, the preeclampsia risk calculator is available free of charge on the FMF website (https://fetalmedicine.org/website/#/calculators accessed on 27 June 2026) and on the FMF mobile, and desktop.
(2) MAP is measured in both arms with strict adherence to protocol [58].
(3) UtA-PI is obtained using a standardized protocol during the first-trimester scan [59].
(4) PlGF concentration is measured using the same automated analyzers employed for PAPP-A in routine Down syndrome screening [15].

Automated analyzers of PlGF and BP monitors must undergo regular calibration to ensure the accuracy of the measurements of biomarkers. In general, there is a certain amount of natural variability (background noise) around an established value (target). This variability is due to “chance or common causes”, which cannot be avoided. However, sometimes a process might be affected by causes that are not from chance patterns, also known as “special causes”. A process under common causes is considered to be “in control”. In contrast, a process affected by special causes is labeled as “out of control,” and such a process demonstrates a deviation from the target. Generally, one should never expect that a process will be in control forever, and at a particular time, a deviation to an out-of-control state will happen due to sudden special causes [60]. In other words, there will be small but sustained shifts throughout a period of time [60].

**Figure 3:**
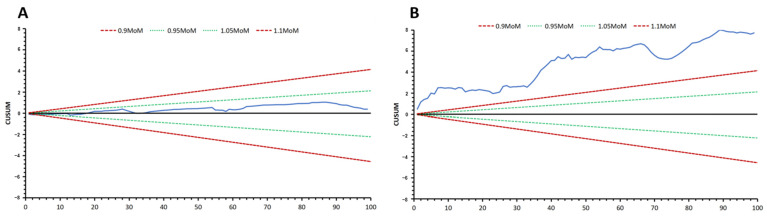
Cumulative sums (CUSUM). (**A**) The measurements are within 0.95 to 1.05 MoM, indicating acceptable accuracy. Black line represents the measurements at 0 MoM; blue line represents sample measurements falling between 0.95 and 1.05 MoM. (**B**) The overmeasurement requires biomarker acquisition reassessment or a biomarker adjustment factor. CUSUM, cumulative sum; MoM, multiples of the median. Black line represents the measurements at 0 MoM; blue line represents sample measurements greater than 1.1 MoM.

**Figure 4:**
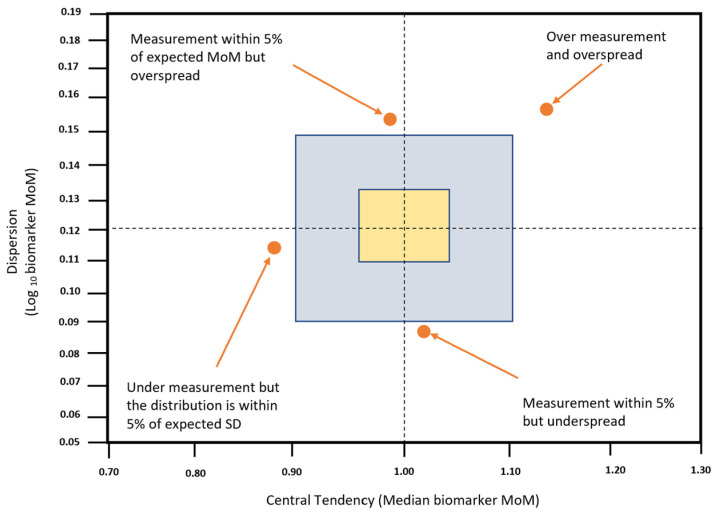
Target plot. The target plot is a common tool to evaluate central tendency (deviation from expected median MoM) and dispersion (deviation from expected median SD).

In prenatal care, early advances in quality control originated from the fetal nuchal translucency measurements for the screening program for Down syndrome. This is the consequence of a significant variation in screening efficiency between centers, illustrating that the conditions under which the measurement is performed should be clearly defined. This approach has led to both quantitative and qualitative forms of control. The quantitative approach compares the distributions of measurements taken against reference values. A qualitative control mechanism evaluates an individual’s performance through an audit process. Such an audit involves both assessing the distribution of measurements for a given sonographer and examining the quality of random images from each sonographer. If the criteria are met, the sonographer will receive a certification of competence and a license to practice until the next audit [61]. The use of quality assurance tools, cumulative sums (CUSUM, Figure 3), and target plots (Figure 4) has been used as quality control for fetal nuchal translucency measurement [57] and serum biochemical markers in Down syndrome screening.

Standardized measurement protocol and quality assurance are important in the context of screening for preeclampsia, as biophysical and biochemical markers are susceptible to inaccurate measurements, which can affect the risk assessment provided to the patients and diminish the overall performance [62].

The biophysical markers, such as MAP and UtA-PI, are equally prone to significant measurement variability, mainly resulting from poor adherence to standardized protocols. Personnel involved in each part of the screening process shall be responsible for the quality assessment of CUSUM and target plots. Measurements of MAP and PlGF are obtained using automated devices that undergo regular calibration [63]. Whenever there is any risk of deviation from the standardized protocol, such as involving new healthcare providers in history taking, MAP, UtA PI, PlGF measurement, or data entry, or when switching to a new batch of reagents or introducing a new analyzer, a supplementary quality assessment should be considered alongside the routine quality assessment. Quality assessment should be included as a part of the service provided by the laboratory responsible for issuing the screening report.

The quality control of the UtA-PI measurement using CUSUM and target plots has demonstrated that sonographers who receive feedback performed better, with results more closely matching the expected measurement distribution than those without receiving any feedback [62,63]. In addition, the non-feedback group had a significantly higher screen-positive rate (6.8% vs. 4.3%; *p* = 0.012) compared to the feedback group [63]. Technical factors contribute to measurement inaccuracies. Other studies have shown that measurements taken too distally from the correct sampling site [59] result in lower pulsatility index values [64]. Operators with a low central tendency (median MoM < 0.95) may be sampling the uterine artery too distally in a significant proportion of patients. Conversely, while not formally tested, it is suspected that operators who tend to overestimate the MoM (>1.05) may be sampling from a site proximal to the recommended site. Similarly, inaccurate biochemical marker results may be due to changes in the batch of reagent used, temperature variation [57], and deviations from the manufacturer’s protocol, or a failure to maintain a continuous quality control process.

## 3. Second and Third Trimester Screening for Preeclampsia

While first-trimester screening models are effective in predicting and preventing preterm preeclampsia, it is less effective for predicting late-onset preeclampsia. The term preeclampsia, as the most common type of preeclampsia, contributes significantly to its associated morbidity and mortality. To date, no consensus exists regarding how to integrate the first-trimester screening together with appropriate prevention and management strategies in later stages of pregnancy (during the second and third trimesters).

There was evidence supporting the role of biomarkers such as PlGF and sFlt-1/PlGF, measured in the second and third trimester, for identifying women who will develop preeclampsia. When women present with symptoms suspected of preeclampsia, a systematic review has demonstrated that angiogenic markers (PlGF and the sFlt-1/PlGF ratio) may be helpful for predicting adverse maternal outcomes [65]. A prospective study including 40,241 women assessed at 19 to 23 weeks reported that the incidence of subsequent PE varied markedly depending on the criteria used, from a low incidence (2.6%) using the traditional definition to a relatively higher rate (3.8%) with the ISSHP-maternal factors + PlGF definition [66]. Additionally, among women presenting with suspected PE at <35 weeks’ gestation, a low PlGF level (≤100 pg/mL) identified those who ultimately develop PE with 76% sensitivity, 69% specificity, and 53% negative predictive values [67]. There is also evidence supporting that low levels of PlGF may be predictive of the development of preeclampsia within 14 days among women presenting symptoms of preeclampsia. A PlGF level below the 5th percentile has a high sensitivity of 0.96 (95% CI, 0.89–0.99) and a negative predictive value of 0.98 (95% CI 0.93–0.995) for preeclampsia within 14 days [68].

Currently, no single test is effective for predicting PE in the second trimester. In a study involving 3529 pregnancies, maternal factors alone, UtA-PI alone, and MAP alone, measured at 22–24 weeks’ gestation demonstrated detection rates of 30.0% (95% CI 10.0–55.0%), 95.7% (95% CI 78.0–99.3%), and 65.2% (42.7–83.6%) for the prediction of early preeclampsia (with delivery before 34 weeks), and 34.6% (95% CI 24.2–46.2%), 41.0% (95% CI 30.0–52.7%), and 39.7% (95% CI 28.8–51.5%) for the prediction of late preeclampsia (with deliveries at or after 34 weeks, at a 10% FPR [69]). Similarly, a study involving 30,775 singleton pregnancies found that UtA PI > 95th centile occurred in 77.2%, 35.9%, and 21.9% of cases with preeclampsia delivered before 34 weeks, 34–37 weeks and after 37 weeks, respectively [70]. The pathogenesis of preeclampsia is attributed to insufficient trophoblastic invasion of the spiral arteries, compromising the normal structural remodeling. The UtA PI findings support a spectrum of impaired placentation, with more extensive impairment of the placenta for cases with early-onset preeclampsia and milder forms of impairment for those with late-onset preeclampsia. For asymptomatic women identified as high-risk following first-trimester screening, there is some evidence supporting the role of UtA-PI measurement at 18–22 weeks and 24–28 weeks of gestation for further stratifying the risk of preeclampsia and SGA [71]. Bonacina et al. reported that in a cohort of high-risk women for preeclampsia identified at first-trimester screening, mean UtA-PI at 18–22 and 24–28 weeks achieved comparably good performance in excluding the subsequent development of preterm preeclampsia and preterm SGA, demonstrating a negative predictive value of >97% for predicting preterm preeclampsia and preterm SGA. It has been proposed that for women who screened high-risk of preeclampsia, the risk of preeclampsia and SGA could be re-assessed through UtA-PI measurements at 18–22 weeks’ gestation and/or at 24–28 weeks’ gestation [72].

A more effective approach for predicting preeclampsia is the model that combines maternal history with measurement of blood pressure and UtA-PI in the second trimester. Using multiple regression analysis, the combined screening including maternal factors, UtA PI, and MAP achieved detection rates of 100% (95% CI 85–100%) and 56.4% (95% CI 40.9–64.0%) for predicting early and late preeclampsia [69]. As an alternative approach to mid-trimester risk stratification for preeclampsia, Litwinska et al. have demonstrated an incremental improvement in screening performance at 19–24 weeks’ gestation, for preeclampsia with delivery at <28, <32, and <36 weeks’ gestation when PlGF and sFlt-1 measurements are added to the combination of maternal factors, UtA-PI and MAP. However, it is noted that the screening performance for the prediction of preeclampsia with delivery at ≥36 weeks’ gestation is suboptimal, with a detection rate of 33.2% (95% CI 30.2–36.3%) [72], highlighting the necessity for risk reassessment at 36 weeks’ gestation to better predict term preeclampsia. For term preeclampsia, a recent multicenter prospective study involving 29,677 pregnancies reported that the FMF triple test, which combined maternal risk factors, MAP, PlGF, and sFlt-1, at 35–37 weeks’ gestation, achieved a detection rate of 79% in the prediction of preeclampsia, at a 10% FPR, with an AUC of 0.923 [73].

For predicting preeclampsia in the second or third trimester, a meta-analysis compared the predictive performance of different models, including PlGF alone, sFlt-1/PlGF ratio alone, and combined models that include PlGF and maternal factors with or without other biomarkers (referred to as PlGF-based models). This study has demonstrated that a combined model including PlGF and maternal factors ± other biomarkers is the most effective method for identifying women at risk of early-onset preeclampsia when used in the second trimester, and better than serum PlGF alone, but similar to the sFlt-1/PlGF ratio for predicting preeclampsia when used in the third trimester [74]. Overall, there remains a paucity of studies investigating the prediction and risk stratification of preeclampsia in the second and third trimesters, and more research is needed to determine the optimal, cost-effective strategy to detect and monitor high-risk women.

A study including 1604 high-risk women (of whom 133 developed preeclampsia) and 403 low-risk women demonstrated that high-risk women who developed preeclampsia presented an increase in MAP throughout pregnancy, an increase in UtA-PI, a reduction in PIGF in the second and third trimesters, and a rise in sFlt-1 in the third trimester compared with those who did not develop preeclampsia despite low-dose aspirin prophylaxis [75]. Additionally, high-risk women who developed preeclampsia exhibited lower levels of mean Z-scores of fetal growth parameters and amniotic fluid index, along with higher values of umbilical artery PI at the mid and third trimesters, compared to high-risk women without preeclampsia and low-risk women [76]. These findings provide further evidence that women screened as high risk of preeclampsia based on first-trimester screening need to be followed up regularly throughout pregnancy. However, the optimal way to follow up with these high-risk women with aspirin prophylaxis remains unclear, and cost-effective protocols for their ongoing surveillance still need to be established. A recent open-label, adaptive, randomized controlled trial (PREVENT-PE) randomized 4037 (49·9%) and 4057 (50·1%) women in the intervention and the control groups, respectively. Preeclampsia risk assessment together with risk-stratified planned early-term birth for those with preeclampsia risk ≥ 1 in 50, assessed at 35^+0^ to 36^+6^ weeks, was performed in the intervention group, while usual care at term was offered for the control group. This trial demonstrated that implementing a risk-stratified early-term delivery strategy for preeclampsia prevention reduced the rate of preeclampsia, with no concomitant rise in emergency cesarean section or neonatal care unit admission [77]. There is emerging evidence supporting the use of FMF preeclampsia screening for the prediction of fetal compromise following induction of labor [78], preterm birth [79], and adverse outcome [80].

## 4. Preventive Strategies and Clinical Implications

A meta-analysis including 45 studies has demonstrated that low-dose aspirin (50–150 mg/day) initiated at or before 16 weeks is associated with a greater reduction in preeclampsia, compared to when initiated after 16 weeks [81]. In addition, a systematic review and meta-analysis have shown that there is a dose–response effect when low-dose aspirin is initiated at or before 16 weeks, while such a dose–response effect is not present when aspirin is initiated after 16 weeks [82]. The Aspirin for Evidence-Based Preeclampsia Prevention (ASPRE) trial, a double-blind, placebo-controlled trial, demonstrated that low-dose aspirin prophylaxis (150 mg per day), from 11 to 14 weeks of gestation until 36 weeks of gestation, was associated with a 62% reduction in preeclampsia in women at high-risk for preterm preeclampsia (odds ratio 0.38, 95% CI 0.20–0.74, *p* = 0.004) [9]. Following this successful trial, it is recommended that women screened as high risk of preeclampsia receive low-dose aspirin (at a dosage between 100 and 150 mg/day) before 16 weeks of gestation, according to key international groups such as the FIGO, the International Society for the Study of Hypertension in Pregnancy, and the International Society of Ultrasound in Obstetrics and Gynecology [7,14]. Given that aspirin may result in increased risk of peripartum bleeding, the Detection of False Positives From First-trimester Preeclampsia Screening at the Second-trimester of Pregnancy (StopPRE) Trial, an open-label, randomized, noninferiority trial demonstrated among women at high risk of preeclampsia with a sFlt-1/PlGF ratio ≤ 38, aspirin discontinuation at 24 to 28 weeks was noninferior to aspirin continuation for prevention of preterm preeclampsia, with a 1.48% and 1.73% incidence of preterm preeclampsia in the intervention and control groups, respectively [83]. This trial demonstrated no beneficial reduction in the risk of major antepartum hemorrhage (hemoptysis and digestive and/or vaginal bleeding) or postpartum hemorrhage ≥ 1000 mL when aspirin was discontinued at 24 to 28 weeks compared to aspirin continuation until 36 weeks, although aspirin discontinuation might reduce the risk of minor bleeding complications (nose and/or gum bleeding) [83]. It is noted that recruitment ceased after an interim analysis of 560 cases, with 968 participants recruited out of the planned sample size of 1080.

In addition to aspirin prophylaxis, there are other strategies to prevent preeclampsia. For individuals with low dietary calcium, the World Health Organization (WHO) and the ACOG recommend calcium supplementation of 1.5–2 g per day to reduce the risk of preeclampsia [84,85]. The latest Cochrane review has refuted the importance of calcium, reporting that calcium makes no difference in preventing preeclampsia compared to a placebo [86]. There are increasing reports on the role of metformin in reducing preeclampsia. Regarding metformin as a prevention of preeclampsia, a trial that evaluated metformin versus placebo offered to women with preterm preeclampsia demonstrated that the median time from randomization to delivery for women who continued to take the trial drug at any dose was 17.5 days in the metformin group, compared to the 7.9 days in the placebo group (a median difference of 9.6 days). This trial has demonstrated proof of concept that metformin has the potential to prolong gestation in women with preterm preeclampsia [87]. A placebo-controlled trial aiming to examine the effect of metformin in the reduction of neonatal birthweight for obese women without diabetes found that there was a 76% reduction in the rate of preeclampsia in the metformin group, compared to the placebo group [88], which highlighted the potential protective effect of metformin in preventing preeclampsia. There is also evidence supporting the benefit of exercise in reducing the risk of preeclampsia [89,90].

## 5. Conclusions

As a major cause of morbidity and mortality for both mothers and babies, significant efforts have been made to refine strategies for predicting preeclampsia. Traditional checklist-based screening from guidelines, such as NICE and ACOG, has limited predictive performance and is considered insufficient for effective preeclampsia screening. Currently, the most effective method for identifying high-risk women for preterm preeclampsia in the first trimester is the FMF triple test, a combined screening that includes maternal factors, MAP, UtA-PI, and PlGF measurements. While effective in predicting preterm preeclampsia in the first trimester, this test performs suboptimally in the prediction of term preeclampsia. Further risk stratification in the second and third trimesters may be achieved with promising competing risk models: a second-trimester model that combines maternal factors, UtA-PI, MAP, and PlGF, along with sFlt-1, and a third-trimester model that incorporates maternal factors, PlGF, and sFlt-1. Advancing the discovery of novel biomarkers may enhance preeclampsia screening, facilitating individualized risk prediction and personalized pregnancy care.

## Data Availability

No new data were created or analyzed in this study. Data sharing is not applicable to this article.
